# The Effects of SJP-001 on Alcohol Hangover Severity: A Pilot Study

**DOI:** 10.3390/jcm9040932

**Published:** 2020-03-31

**Authors:** Joris C Verster, Thomas A Dahl, Andrew Scholey, Jacqueline M Iversen

**Affiliations:** 1 Division of Pharmacology, Utrecht Institute for Pharmaceutical Sciences (UIPS), Utrecht University, 3584CG Utrecht, The Netherlands; 2 Institute for Risk Assessment Sciences (IRAS), Utrecht University, 3584CM Utrecht, The Netherlands; 3 Centre for Human Psychopharmacology, Swinburne University, Melbourne VIC 3122, Australia; andrew@scholeylab.com; 4 Sen-Jam Pharmaceutical, 223 Wall St., #130, Huntington, NY 11743, USA; tadahl@sen-jam.com (T.A.D.); jackie@sen-jam.com (J.M.I.)

**Keywords:** alcohol, hangover, treatment, prevention, SJP-001, naproxen, fexofenadine

## Abstract

Background. Despite a clear market need and many hangover products available, currently there is no hangover treatment that is supported by substantial scientific evidence demonstrating its efficacy and safety. A pilot study was conducted to investigate the effects of a potential new hangover treatment, SJP-001, and its constituents (220 mg naproxen and 60 mg fexofenadine) on hangover severity. Methods. *N* = 13 healthy social drinkers (36.3 ± 8.9 years old) participated in a double-blind, factorial design, cross-over study. On each test day, they consumed their own choice of alcohol up to a self-reported level sufficient to elicit a next-day hangover. Treatments were administered prior to onset of drinking. Next morning, hangover severity was assessed with the Acute Hangover Scale (AHS). Subjects were included in the efficacy analysis only if they reported a hangover after placebo. Results. *N* = 5 subjects (60% male, 35.2 ± 9.0 years old) were included in the analysis. They consumed a mean (SD) of 4.6 ± 1.1 units of alcohol and had an average peak breath alcohol concentration (BrAC) of 0.065% across conditions. Compared to placebo, SJP-001 significantly improved the AHS overall hangover severity score (0.8 ± 0.3 versus 1.5 ± 0.9, *p* = 0.042). Compared to placebo, SJP-001 also reduced scores on the individual item ‘hangover’, although the observed improvement (−1.6) did not reach statistical significance (*p* = 0.102). The differences from placebo after naproxen alone and fexofenadine alone were not statistically significant. SJP-001 also improved scores for the individual hangover symptoms tired, thirsty, headache, dizziness, nausea, and loss of appetite, but these effects did not reach statistical significance. Discussion. Compared to placebo, SJP-001 significantly reduced overall hangover severity. The effects of SJP-001 should be further examined in a double-blind, placebo-controlled trial with a larger sample size and controlled administration of sufficient amounts of alcohol to provoke a more substantial alcohol hangover.

## 1. Introduction

The alcohol hangover is defined as “the combination of negative mental and physical symptoms which can be experienced after a single episode of alcohol consumption, starting when blood alcohol concentration (BAC) approaches zero” [1,2]. Recent research suggests that there is no BAC threshold for producing hangover, but that hangovers may occur at all BAC levels, and are most likely elicited following consumption of more alcohol than usual on a drinking occasion [3,4]. 

Alcohol hangovers are typically characterized by a combination of symptoms, affecting mood, cognition, and physical functioning [5,6,7,8]. These negatively impact daily activities including, but not restricted to, job performance [9] and driving [10,11,12]. In the USA, the annual economic costs of alcohol hangover in terms of absenteeism and presenteeism have been estimated at $173 billion annually [13]. A recent UK study rated the annual economic costs of hangover at 4 billion GBP [14]. Unsurprisingly, consumers have expressed a clear need for an effective hangover treatment [15]. However, to date, little research has been devoted to the development of effective and safe hangover treatments, and currently there is no evidence-based hangover treatment [16,17,18]. The pathology of alcohol hangover is not yet elucidated [19,20,21], although alcohol metabolism and the immune response to alcohol consumption are current research foci [22,23,24,25].

The immune system likely plays a role in the development of alcohol hangover, with significant immune reactions (e.g., changes in blood cytokine levels and C-reactive protein) being associated with heavy alcohol consumption and subsequent hangovers [23,26,27,28,29,30]. In addition, alcohol and acetaldehyde liberate histamine from mast cells and depress histamine elimination by inhibiting diamine oxidase [31]. Independently of alcohol consumption, prostaglandin and histamine release contributes to inflammation, pain (including headache), and fatigue [32,33,34]. These are all symptoms of alcohol hangover [5,6]. Thus, it is hypothesized that an intervention that prevents the release of prostaglandin and histamine may serve as an effective treatment to prevent alcohol hangover.

SJP-001 has been developed as such a potential new treatment for prevention of alcohol hangover. SJP-001 is a combination of two over-the-counter (OTC) oral generic drugs (i.e., a nonsteroidal anti-inflammatory drug (220 mg naproxen) and an H_1_-antagonist (60 mg fexofenadine)). Both naproxen and fexofenadine are individually marketed in USA as over-the-counter (OTC) drugs [35,36]. The anti-inflammatory properties of naproxen are well documented [37]. Although prescribed primarily for its H_1_ antagonist activity [38], fexofenadine also exhibits some anti-inflammatory properties by modulating the release of a variety of proinflammatory mediators. For example, in a study to evaluate the immunomodulatory properties of antihistamines, it was found that fexofenadine downregulated IL 4–induced production of IL 5 and suppressed IL 12–induced secretion of IFN γ [39]. 

Given this background, a pilot study was conducted to determine if SJP-001 can reduce hangover severity when taken prior to drinking in a sample of healthy social drinkers. 

## 2. Methods

The study followed a randomized, double-blind, placebo-controlled, factorial, cross-over design. In addition to a screening day, there were four study visits, separated by a washout period of three to nine days. The study was sponsored by Sen-Jam Pharmaceutical (JMI Capital Group) and conducted by Clinilabs, Inc. in accordance with the guidelines of the Declaration of Helsinki and its latest amendments. Ethics approval was obtained from the Chesapeake Institutional Review Board (Study number: Pro00016219) and written informed consent was obtained from all subjects. Data from the study are on file (Sen-Jam Pharmaceutical) and available upon reasonable request. 

### 2.1. Subjects

*N* = 16 self-reported moderate drinkers that previously experienced alcohol hangovers were recruited via online advertisement. Screening procedures included a brief physical examination including height and weight, collection of demographic information, medical history and vital signs, review of prior and concomitant medications, and a urine drug and pregnancy screen, and a breathalyzer alcohol test. To be included, subjects had to be nonsmoking men or women between 25 and 65 years old, have a body mass index (BMI) between 19 and 32 kg/m^2^, have a regular, habitual bedtime between 21:30 and 24:00 h, and have a good general health as determined by a thorough medical history and physical examination including vital signs, conducted by the study physician. Subjects had to be self-reported moderate drinkers of alcohol, which was approximated with a breath alcohol concentration (BrAC) of 0.04%–0.11% on usual drinking occasions (corresponding to 2 to 5 or 3 to 7 alcoholic drinks for a 70 kg female and male, respectively, over a 2 to 3 h period). Subjects were included if they reported that this amount of alcohol usually resulted in a next-day hangover. Subjects were excluded if they reported acute illness within 14 days prior to screening visit, experienced an allergic reaction or upper respiratory tract infection within 7 days of screening visit, had been vaccinated within 7 days of screening visit, had a history of clinically significant allergies (except for untreated, asymptomatic, seasonal allergies at time of dosing), hematological, renal, endocrine, pulmonary, gastrointestinal, cardiovascular, hepatic, or neurological disease, cancer or diabetes, or psychiatric illness, previous or current Substance-Related Disorder as defined by DSM-5, self-reported usual consumption of more than 14 units of alcohol per week, recent (within one month) or current use of tobacco or nicotine products. In addition, subjects were excluded if medical examination revealed a clinically significant, unstable medical illness, a positive alcohol breathalyzer or urine drug screen test (including cocaine, THC-marijuana, opiates, amphetamines, methamphetamine, phencyclidine, benzodiazepines, barbiturates, methadone, MDMA-ecstasy, oxycodone, and propoxyphene) which was provided at screening, when subjects had a blood pressure >140/90 mm/Hg or heart rate >100 bpm. Women who were pregnant or breastfeeding, or had a positive urine pregnancy test at screening were also excluded. Subjects taking any prescription or OTC oral pain medication(s) or antihistamine drug, or who previously experienced an allergic reaction or adverse event associated with aspirin, nonsteroidal anti-inflammatory drugs (NSAID), or antihistamine usage were excluded. Finally, subjects were excluded if they were unwilling to forgo caffeine consumption with or following dinner on each treatment night or who were unwilling to comply with study restrictions for prohibited medications/foods throughout study participation.

### 2.2. Procedures

During the four test days, each participant arrived at the clinical unit at approximately 5 pm. On the first test day, subjects were randomized to one of four treatment sequences according to Latin Square assignment. On test days, after general health assessments, including a urine drug and pregnancy test and a breathalyzer test to ensure a BrAC of zero, subjects were served a standardized dinner. Alcohol was available with dinner. Subjects received an oral dose of either SJP-001 (220 mg naproxen and 60 mg fexofenadine), naproxen alone (220 mg), fexofenadine alone (60 mg), or placebo. Treatments consisted of two oral capsules administered 30 min (first capsule) and 15 min (second capsule) before the start of alcohol consumption, taken with approximately 240 mL of water. Treatments were self-administered by subjects under the supervision of study personnel. 

Subjects remained together in a lounge room during dinner and thereafter for the evening. They were permitted to socialize, read, and watch television. Study staff were present to monitor alcohol and food consumption and general behavior. Foods high in histamine such as red meat, lamb, and aged cheese were not served. Subjects were provided a variety of alcoholic beverages from which to choose including red and white wine, various types of beer, champagne, and liquors. Water was provided, along with other nonalcoholic beverages as mixers. They were instructed to consume the amount of alcohol (and nonalcoholic drinks including water) that had in the past resulted in a hangover, and consume the same type and amount of alcohol on each test day. For example, if a subject reported at screening that consuming four glasses of red wine in a 3 h period had previously resulted in a hangover, the target for that subject was to consume four glasses of red wine at each visit. Subjects could mix different types of alcohol and were allowed to drink at their own pace; all alcohol consumption was completed within a maximum 3 h period. Light snacks (e.g., pretzels, potato chips, nuts) were provided during the alcohol consumption period. At the end of alcohol consumption, a breathalyzer test was conducted to assess BAC. Subjects stayed overnight in the study center. They were required to go to bed at their usual habitual bedtime. Subjects slept in a private room with a bed, night table, writing table, and chair, and were instructed to remain in bed for 8 h with the lights turned off. Subjects were allowed to deviate from these instructions to use the restroom during the night. Study staff made rounds during the night to monitor subjects’ safety. Next morning, if necessary, subjects were awakened 8 h after their bedtime. Within 10–20 min after awakening, and prior to breakfast or the consumption of any coffee or other caffeine-containing beverages, a breathalyzer test and brief neurological assessments (tests examining walking and heel-to-toe walking, and the Romberg test to evaluate balance) were conducted. A postsleep questionnaire was completed, including questions regarding total sleep time, sleep onset latency, number of nightly awakenings, time awake while in bed, and a rating of sleep quality on a scale from 1 (poor) to 10 (excellent). Next-morning hangover severity was assessed with the Acute Hangover Scale [40]. Thereafter, subjects received breakfast and the test day was ended.

### 2.3. Assessment of Hangover Severity

The Acute Hangover Scale [40] consists of nine items including ‘hangover’, ‘thirsty’, ‘tired’, ‘headache’, ‘dizziness/faintness’, ‘loss of appetite’, ‘stomachache’, ‘nausea’, and ‘heart racing’, which could be rated on a scale ranging from 0 to 7. The anchors of the Likert-type scale were ‘none’ (score of 0), ‘mild’ (score of 1), ‘moderate’ (score of 4), and ‘incapacitating’ (score of 7). Overall hangover severity was computed by calculating the average score across the AHS nine items. In the interest of safety, subjects with hangover symptoms were confined to the study center at the discretion of the clinician until symptoms had improved.

### 2.4. Statistical Analysis

Subjects were included in the statistical analysis only if they reported a hangover on the placebo test day. The reasons for this were twofold. Firstly, the absence of a hangover in the placebo condition implies that the subject had not complied with the instructions to drink to levels which would typically produce hangover. Secondly, we wished to evaluate if the combination of naproxen and fexofenadine in SJP-001 was superior to either compound alone. This required us to compare effects on a hangover in the placebo group (note that the absence of a hangover in any of the nonplacebo arms could theoretically be attributable to efficacy of the treatment so such cases could not be excluded). Statistical analyses were conducted with SPSS (IBM Corp. Released 2013. IBM SPSS Statistics for Windows, Version 25.0, IBM Corp. Armonk, NY, USA). Mean and standard deviation (SD) were computed for all variables. The primary outcome measure of the study was the average AHS score. Secondary outcomes were the individual AHS items. Overall hangover severity and individual symptom ratings after SJP-001 and placebo were compared applying a nonparametric Related-Samples Wilcoxon Signed Rank test.

## 3. Results

Of the *N* = 16 subjects that were screened, *N* = 13 met all inclusion and exclusion criteria and participated in the study. Seven subjects who reported no hangover on the placebo test day were excluded from the statistical analysis (see previous section). Another subject was excluded due to significant sleep difficulties in the clinical setting. *N* = 5 subjects were included in the final dataset. For one of these subjects, the last test day (naproxen) was discontinued due to noncompliance with study procedures during the drinking session. The demographic data of the included subjects are summarized in Table 1.

In the study, subjects consumed on average 4.6 alcoholic drinks and had an average BrAC of 0.065% at the end of the drinking session. BrACs did not statistically differ between conditions (see Table 2). After the drinking session, subjects slept in the clinical research unit. Table 2 summarizes the sleep outcomes for each treatment condition. No significant differences were found between SJP-001 and placebo.

**Figure 1:**
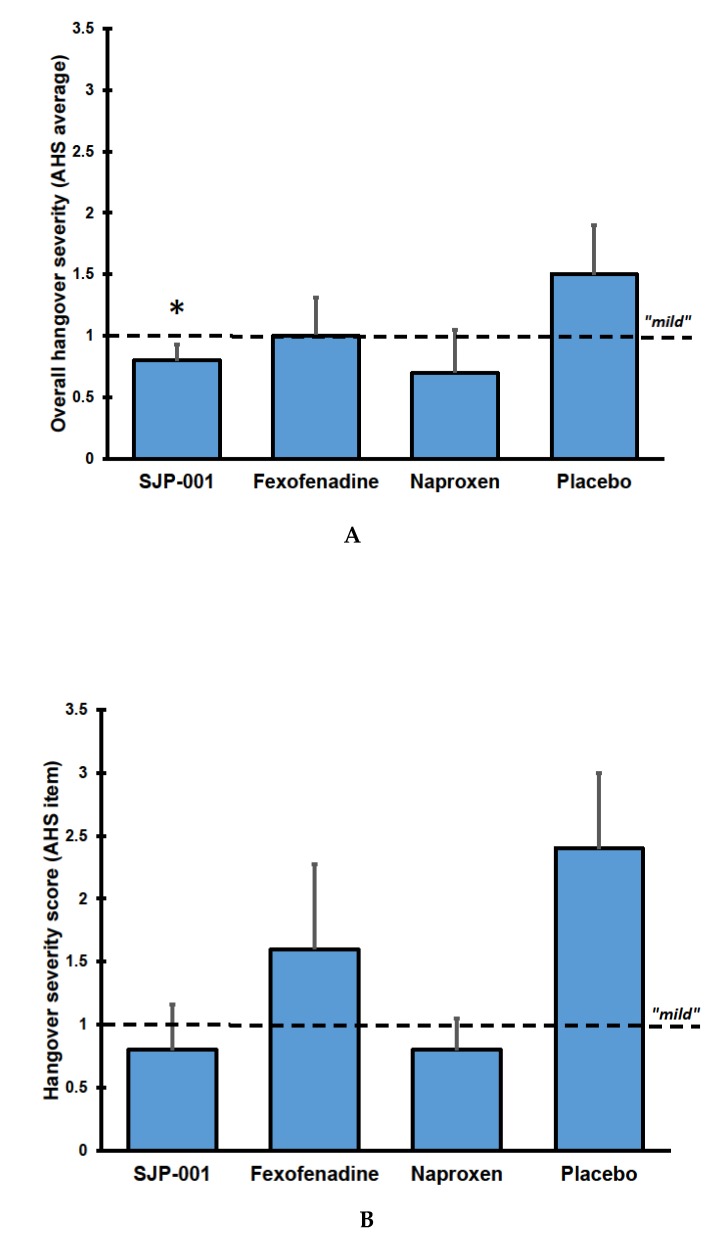
Overall hangover severity by group. (**A**) shows the average AHS scores; (**B**) shows the scores on the individual AHS item ‘hangover’. Mean scores with standard errors are shown. Abbreviation: AHS = Acute Hangover Scale. Significant differences (*p* < 0.05) between the treatments and placebo are indicated by *.

Mean ± SD AHS scores were mild and equaled 1.5 ± 0.9 after placebo, 0.8 ± 0.3 after SJP-001, 1.0 ± 0.7 after fexofenadine, and 0.7 ± 0.7 after naproxen. Scores on individual hangover symptoms are listed in Table 3. Compared to placebo, SJP-001 significantly improved overall hangover severity (*p* = 0.042), whereas the differences from placebo after naproxen (*p* = 0.066) and fexofenadine (*p* = 0.345) were not statistically significant (See Figure 1A).

Also, compared to placebo, SJP-001 reduced scores on the individual item ‘hangover’, although the difference of −1.6 did not reach statistical significance in this small sample (*p* = 0.102) (see Figure 1B). SJP-001 also improved scores for other individual hangover symptoms including tired, thirsty, headache, dizziness/faintness, nausea, and loss of appetite (none of the differences between SJP-001 and placebo reached statistical significance). After fexofenadine alone and naproxen alone, none of the individual item scores differed significantly from placebo.

## 4. Discussion

The findings of the current pilot study suggest that SJP-001 may be effective in the prevention of alcohol hangover. However, the observations should be interpreted with caution, as this pilot study had a small sample size (albeit using a cross-over design). The observed significant reduction in overall hangover severity, assessed both via the average AHS score and the individual item “hangover”, justifies further investigation of the properties of SJP-001 in future randomized controlled trials (RCTs) with an adequate sample size and improved methodology.

It has been argued that when conducting pilot studies with a small sample size, one should focus on descriptive statistics and estimation rather than formal hypothesis testing to infer whether findings are clinically relevant or not [41,42,43]. To further evaluate descriptive data of the current study, difference scores in hangover severity between SJP-001 and placebo were calculated. These mean (SD) difference scores equal −0.64 (0.62) for the AHS overall hangover severity score and −1.6 (1.67) for the one-item hangover severity score. Applying the anchor-based approach [44], both the average AHS and one-item hangover score are between the anchors ‘mild’ and ’moderate’ after placebo which were relevantly improved towards scores between the anchors ‘none’ and ‘mild’ after SJP-001. When applying the one-half SD criterion as a benchmark [45], the minimal clinically important difference (MCID) should be greater than −0.31 on the AHS and greater than −0.84 for the one-item hangover severity score. Thus, both the AHS (−0.64 > −0.31) and the one-item hangover severity difference score (−1.6 > 0.84) can be considered as clinically relevant reductions in hangover severity. However, to infer clinical relevance, the single-item hangover severity score is likely to be more accurate with regard to clinical relevance than the AHS aggregate symptoms score [46]. Taken together, evaluating descriptive data further supports the decision to conduct adequately powered RCTs to enable more definitive conclusions regarding the efficacy of SJP-001.

Turning to individual symptoms, it may be argued that the effects of SJP-001 are relatively similar to those observed when naproxen alone is administered. Importantly, for the AHS scores, the difference between SJP-001 and placebo (but not naproxen and placebo) is statistically significant. This is likely due to the fact that, although the mean AHS scores of SJP-001 and naproxen are similar, there is a considerable difference in the corresponding standard deviation of SJP-001 and naproxen (0.3 versus 0.7, respectively). One could argue that the lower variance after SJP-001 in contrast to naproxen alone is a result of the naproxen–fexofenadine combination resulting in a more stable, less variable treatment effect due to the anti-inflammatory properties of fexofenadine. Also, the achieved BrAC after SJP-001 was higher than after naproxen (0.064% versus 0.053% BrAC, respectively), which may have resulted in an enhanced treatment effect after naproxen compared to that of SJP-001. Had equal BrACs been achieved, the effect of SJP-001 may have been superior to naproxen alone. Alternatively, it may be that the observed difference in standard deviation reflects the small sample size of this pilot study, and will not be replicated in a larger, well-powered RCT. Future research should investigate these hypotheses.

In addition to assessing initial evidence regarding the effectiveness of SJP-001 in preventing hangover, an important goal of this pilot study was to verify whether the current study design is suitable for large sample, double-blind RCTs. From the current pilot study, several important lessons were learned regarding limitations to the study design.

Firstly, the sample size of this pilot study was small. Future RCTs should be adequately powered. An important reason for this small study size was that of the *N* = 13 subjects that completed the study, only *N* = 5 were suitable to be included in the data analysis. Although subjects consumed the self-reported amount of alcohol that was sufficient to induce hangover, seven subjects reported to have no hangover in the placebo condition. In future RCTs, the amount of alcohol to provoke a hangover should be determined in more detail to prevent such a large dropout. Currently, a hangover sensitivity scale is in development to aid this process. This approach will increase the likelihood that a hangover will be present on each test day.

The exclusion of subjects who had no hangover following placebo only was necessary to allow meaningful statistical comparison across the groups. It is possible that excluding individuals not experiencing hangover in the placebo condition may have somehow increased the probability of a significant outcome favoring SJP-001. If this were the case, however, one might also expect significant effects when comparing the naproxen-only and fexofenadine-only arms with placebo. To verify this, we conducted two additional analyses, in which we compared naproxen-only versus placebo and fexofenadine-only versus placebo, including only subjects with a placebo score greater than zero. Again, no significant differences between the treatments were found for the AHS and one-item overall hangover severity score. Therefore, we believe that excluding subjects without a hangover in the placebo condition did not affect the probability of obtaining a significant outcome favoring SJP-001.

A second issue in this pilot study is that subjects could consume their preferred types of alcohol. Although the type(s) of drink(s) were the same on each test day, eliminating intraindividual differences, this does introduce interindividual variability. Additionally, alcoholic drinks have variable amounts of congeners and histamines, which may impact study outcomes. A high congener content has been shown to negatively impact hangover severity [47]. One obvious solution to this is to administer one standard type of alcoholic drink to all subjects. On the other hand, this may not adequately reflect participants’ usual drinking behavior. Achieving a balance between experimental control and ecological validity is a challenge for hangover research [48].

One subject was excluded because of experiencing significant sleep difficulties in the clinical research unit. Sleeping in a new (and especially clinical) environment may require adaptation. The current study did not include a sleep rehearsal night during screening to identify possible ‘first-night effects’ or other sleep disturbances [49,50]. It is advisable to include a sleep rehearsal night in future RCTs to screen for subjects with possible sleep disturbances. Further, the current study used subjective measures of sleep. Subjective assessments of sleep do not always correspond to objective sleep measures [51], such as polysomnography or actigraphy. Future RCTs could usefully include objective sleep measures, for example, by using mobile technology such as the GENEactiv device [51].

Finally, the assessment of alcohol hangover depends solely on self-report. The most direct method to assess hangover severity is a single question allowing subjects to rate their hangover severity, for example, a 0 to 7 score as used for the AHS [40], or a 0 to 10 rating scale ranging from absent to extreme [52]. Since hangover may differ qualitatively from person to person, a single question is more likely to accurately reflect overall hangover severity than an aggregate score of variable hangover symptoms, such as that utilized with current hangover scales [40,53,54]. Aggregate symptom scores may be lower than one-item overall hangover severity scores as the scales may include low-frequency/low-severity symptoms and omit high-frequency/high-severity symptoms [46]. Notably in this pilot study, the single-item hangover score was higher, and better differentiated SJP-001 from placebo than the AHS aggregate symptom score. Relying on self-report to investigate hangovers is necessary, as there is currently no known objective criteria or biomarker for hangover severity. It is, however, important to include biomarkers of immune function in future RCTs, to assess the immune response to alcohol consumption in the placebo condition, and to infer whether SJP-001 is capable of reducing this effect. Such data will be important to provide insight into the mechanism(s) of action of SJP-001.

In conclusion, the data from this pilot study suggest that SJP-001 is effective in preventing alcohol hangover. However, the sample size of this study was very small. Therefore, the effects of SJP-001 should be further evaluated in RCTs with a larger sample size.

## Figures and Tables

**Table 1:** Demographics, morphometrics, and drinking characteristics of the sample.

	Overall	Subject 7	Subject 8	Subject 9	Subject 12	Subject 16
Sex (male/female)	3/2	Male	Male	Male	Female	Female
Age (years)	35.2 (9.0)	27	47	33	27	42
Weight (lb)	170.8 (13.7)	169.4	192.6	172.8	162.4	156.6
Height (in)	67.4 (2.6)	67.5	68.9	70.7	65.5	64.2
BMI (kg/m^2^)	26.4 (1.5)	26.1	28.5	24.3	26.6	26.7
Habitual bedtime ^1^ (h:min)	23:12 (0:34)	00:00	23:30	23:00	22:30	23:00
Preferred alcohol type ^2^	-	Rum	Mixed ^3^	Mixed ^4^	Rum	Vodka
Units alcohol for hangover ^5^	4.6 (1.1)	3	6	5	4	5

Mean and standard deviation (SD) are shown for the overall sample and five individual subjects. ^1^: the individual habitual bedtime was used for each test day. ^2^: these drinks were also consumed on each test day. ^3^: subject consumed white wine (Chardonnay), red wine (Pinot Noir), and tequila on a usual drinking occasion. ^4^: subject consumed Scotch whiskey, beer, and red wine on a usual drinking occasion. ^5^: this amount of alcohol units was also consumed on each test day. Abbreviation: BMI = body mass index.

**Table 2:** Alcohol consumption and sleep outcomes.

	SJP-001	Fexofenadine	Naproxen	Placebo
Units of alcohol consumed	4.6 (1.1)	4.6 (1.1)	4.6 (1.1)	4.6 (1.1)
BrAC (3h) (%)	0.064 (0.034)	0.068 (0.026)	0.053 (0.030)	0.072 (0.038)
Total sleep time (min)	412.0 (45.9)	377.0 (66.3)	406.0 (59.3)	421.0 (44.2)
Number of nightly awakenings	1.6 (1.1)	2.0 (1.4)	1.5 (1.9)	1.6 (1.1)
Sleep onset latency (min)	34.0 (18.5)	52.0 (30.9) *	45.0 (17.3)	12.2 (11.3)
Time awake while in bed (min)	29.4 (30.7)	34.2 (38.8)	13.0 (12.9)	36.0 (38.7)
Sleep quality	7.2 (1.5)	4.8 (3.6)	8.3 (1.0)	7.8 (1.6)

Mean and standard deviation (SD) are shown for alcohol consumption and sleep outcomes in the four treatment conditions. Significant differences (*p* < 0.05) between the treatments and placebo are indicated by *. Abbreviation: BrAC = breath alcohol concentration.

**Table 3:** Mean Acute Hangover Scale and individual hangover symptom scores.

Symptoms	SJP-001	Fexofenadine	Naproxen	Placebo
Hangover	0.8 (0.8)	1.6 (1.5)	0.8 (0.5)	2.4 (1.3)
Thirsty	2.4 (1.8)	1.6 (1.1)	1.5 (2.4)	2.8 (2.4)
Tired	2.2 (1.9)	2.4 (2.1)	1.8 (2.1)	2.8 (1.6)
Headache	0.4 (0.5)	1.6 (1.5)	0.3 (0.5)	1.6 (2.5)
Dizziness/faintness	0.0 (0.0)	0.2 (0.4)	1.0 (2.0)	1.0 (1.2)
Loss of appetite	0.4 (0.5)	1.0 (1.7)	0.3 (0.5)	1.0 (0.7)
Stomachache	0.8 (1.3)	0.4 (0.9)	0.3 (0.5)	0.6 (0.5)
Nausea	0.4 (0.9)	0.2 (0.4)	0.3 (0.5)	1.0 (1.7)
Heart racing	0.4 (0.5)	0.2 (0.4)	0.0 (0.0)	0.2 (0.4)
Mean AHS score	0.8 (0.3) *	1.0 (0.7)	0.7 (0.7)	1.5 (0.9)

Mean (SD) are shown for the AHS and its individual items for the four treatment conditions. Abbreviation: AHS = Acute Hangover Scale. Significant differences (*p* < 0.05) between the treatments and placebo are indicated by *.
